# Astrocytes at the heart of sleep: from genes to network dynamics

**DOI:** 10.1007/s00018-025-05671-3

**Published:** 2025-05-21

**Authors:** Félix Bellier, Augustin Walter, Laure Lecoin, Fréderic Chauveau, Nathalie Rouach, Armelle Rancillac

**Affiliations:** 1 Neuroglial Interactions in Cerebral Physiology and Pathologies, Center for Interdisciplinary Research in Biology-CIRB, Collège de France, CNRS UMR 7241/Inserm U1050, Université PSL, PSL-NEURO, 11, Place Marcelin Berthelot, 75005 Paris, France; 2 IRBA (Institut de Recherche Biomédicale Des Armées), Brétigny-sur-Orge, France

**Keywords:** Adenosine, Astrocyte, Calcium signaling, Gap junctions, Gliotransmission, Metabolism, Prostaglandin D2, Synaptic coverage, Synaptic homeostasis, Tripartite synapse, Ventrolateral preoptic nucleus (VLPO), Excitation-inhibition balance, Synaptic plasticity, Circadian rhythm, Neuroglia, Neurovascular coupling, NREM sleep

## Abstract

Astrocytes have transcended their role from mere structural scaffolds to pivotal regulators of neural circuitry and sleep–wake dynamics. The strategic proximity of their fine processes to blood vessels and synapses positions them as key players in neurobiology, contributing to the tripartite synapse concept. Gap-junction proteins also enable astrocytes to form an extensive network interacting with neuronal assemblies to influence sleep physiology. Recent advances in genetic engineering, neuroimaging and molecular biology have deepened our understanding of astrocytic functions. This review highlights the different mechanisms by which astrocytes regulate sleep, notably through transcriptomic and morphological changes, as well as gliotransmission, whereby intracellular calcium (Ca^2+^) dynamics plays a significant role in modulating the sleep–wake cycle. In vivo optogenetic stimulation of astrocytes indeed induces ATP release, which is subsequently degraded into adenosine, modulating neuronal excitability in sleep–wake regulatory brain regions. Astrocytes also participate in synaptic plasticity, potentially modulating sleep-associated downscaling, a process essential for memory consolidation and preventing synaptic saturation. Although astrocytic involvement in synaptic maintenance is well supported, the precise molecular mechanisms linking these processes to sleep regulation remain to be elucidated. By highlighting astrocytes' multiple roles in sleep physiology, these insights deepen our understanding of sleep mechanisms and pave the way for improving sleep quality.

## Introduction

Astrocytes are a type of glial cells first described in 1858 by the biologist Rudolf Virchow. The term "astrocyte" comes from the Greek *astron*, meaning “star”, and *kytos* for “cell”. This name, chosen in 1895 by Michael von Lenhossèk, to reflect their typical star-like morphology, is reminiscent of celestial bodies [1]. Their extensive processes enable them to establish close contact with blood vessels and neuronal synapses, giving them a highly strategic position. However, recognition of astrocyte functions took time due to several technical and conceptual challenges that hindered their study for decades. First, their highly branched and fine processes made it difficult to analyze their structure and functions using traditional microscopy. Moreover, their lack of electrical excitability led neuroscientists to focus primarily on neurons, which communicate via action potentials. Inadequate experimental tools further delayed progress as early electrophysiological methods were optimized for recording rapid neuronal activity, but were not suited for detecting astrocytic calcium-based signaling. Additionally, the absence of specific molecular markers made it difficult to distinguish astrocytes from other cells types and to manipulate them selectively. It was only with advances in genetic tools (astrocyte-specific transgenic models) and imaging techniques (two-photon microscopy and superresolution microscopy), that the intricate roles of astrocytes started to be uncovered. Historically viewed as passive structural elements, astrocytes are now recognized as active participants in neuronal communication. They express all the molecular machinery to sense synaptically-released neurotransmitters via the expression of a wide repertoire of receptors, transporters, and ion channels. They respond to synaptic activity via calcium (Ca^2+^) signaling [2] and can in turn release gliotransmitters or change their coverage of synapses, which can modulate the diffusion or reuptake of neurotransmitters and thereby regulate neurotransmission. Thus, astrocytes are not merely sensitive to neuronal activity, but can also initiate changes in synaptic activity, placing these cells at the heart of the fine regulation of various behaviors. Remarkably, over a century ago, the pioneering neuroscientist Santiago Ramón y Cajal was the first to propose a dynamic role for astrocytes in regulating sleep. He proposed that astrocytic processes extend into synapses during sleep, acting as a ‘circuit breaker’ to block synaptic transmission and retract during wakefulness to restore it [3]. However, Cajal’s hypothesis was not tied to a specific brain region, serving instead as a general conceptual model.

More recent studies, such as those by Bellesi et al. have provided experimental evidence focusing on the cortex. Using three-dimensional electron microscopy and immunohistochemistry, they demonstrated that astrocytic processes extend toward synapses during wakefulness, and retract during sleep, suggesting a dynamic role for astrocytes in synaptic regulation across the sleep–wake cycle [4]. Using translating ribosome affinity purification technology and microarrays, Bellesi et al. further demonstrated that arousal is accompanied by the upregulation of genes involved in extracellular matrix dynamics and cytoskeleton remodeling. This genetic activation facilitates the elongation of astrocytic processes toward synapses during wakefulness, in contrast to their retracted state observed state during sleep [4]. This reinforces the notion that astrocytes actively modulate synaptic interactions across the sleep–wake cycle.

Interestingly, these cortical-specific dynamics contrast Cajal’s prediction of astrocytic behavior during sleep and wake states. The close proximity of PAPs can enhance glutamate uptake, effectively reducing extracellular concentrations, while their withdrawal may increase glutamate spillover. Thus, the idea of a differential regulation of glutamate proposed by Cajal remains valid, albeit one that may vary across different brain regions and adapt to their specific functional demands.

The proximity of some astrocytic processes to synapses led to the concept of the tripartite synapse [5]. It is now clearly established that astrocytes play essential roles in brain functioning, not only in maintaining neuronal environment and survival, but also in modulating neural networks. They provide structural and metabolic support to neurons, and their proximity to blood vessels allows them to participate in the formation and maintenance of the blood–brain barrier, as well as in neurovascular coupling, adjusting blood flow in response to local neuronal activity [6–8] (Table 1). Astrocytes regulate ionic and aqueous homeostasis, particularly by regulating extracellular potassium levels [9, 10]. They can also trap and neutralize reactive oxygen species [11] and participate in the formation and maturation of synapses [12]. Following brain injury, astrocytes also contribute to the formation of the glial scar and the repair of the nervous tissue [13, 14]. Although not immune cells, astrocytes also play a role in the brain's inflammatory response [15] and phagocytose [16]. Finally, astrocytes communicate with each other via gap junctions, allowing them to form networks covering and modulating neuronal assemblies [17–20]. Thus, astrocytes regulate neuronal circuits underlying rhythmic behaviors such as locomotion, respiration, mastication, gastrointestinal motility, feeding, as well as sleep–wake cycles [21–23].

**Table 1:** Summary table of the main astrocytic functions associated with sleep regulation

Features	Approaches	Effect	Model	References
Astrocytic Ca^2+^ variations	Genetic ablation of IP3R2 signaling pathway, genetically encoded Ca^2+^ indicators and two-photon imaging	Increased astrocytic Ca2 + signals in the neocortex precedes transitions from NREM sleep to wakefulness Genetic ablation of key Ca2 + signaling pathways in astrocytes disrupts NREM sleep	Mouse	Bojarskaite et al. [67]
	Miniature miniscope, Two-Photon microscopy, Polysomnography, sleep deprivation, Calcium imaging, Conditional knockout of STIM in astrocytes	Astroglial calcium levels in the frontal cortex are highest during wakefulness and lowest during sleep Astroglial calcium levels increase with sleep deprivation and decrease during recovery sleep Reducing astroglial calcium impairs the response to sleep deprivation	Mouse	Ingiosi et al. [64]
	Polysomnography, Sleep Deprivation, Chemogenetic	Activation of basal forebrain astrocytes increases wakefulness and reduces REM sleep No compensatory changes in sleep drive	Mouse	Ingiosi et al. [80]
	Optogenetic stimulation of astrocytes in the posterior hypothalamus	Optogenetic stimulation of astrocytes in the hypothalamus promotes sleep (NREM et REM), not SWA	Mouse	Pelluru et al. [73]
	Genetically encoded ratiometric Ca^2+^	Ca^2+^ levels in astrocytes decrease during REM sleep, and increase after the onset of wakefulness. In contrast, differences in Ca^2+^ levels during NREM sleep were observed among the different brain regions, and no significant decrease was observed in the hypothalamus and pons	Mouse	Tsunematsu et al. [71]
	In vivo Two-photon calcium Imaging and Electrophysiological recordings, DREADDs in astrocytes	Gi- and Gq-coupled GPCR signaling in cortical astrocytes separately control NREM sleep depth and duration Astrocytic signaling causes differential changes in local and remote cortical regions	Mouse	Vaidyanathan et al. [68]
Brain clearance	Two-photon Microscopy, Fluorescent Tracer Injections, Aqp4-null mice	Astrocytic AQP4 is crucial in regulating this fluid movement and maintaining convective currents that help clear interstitial solutes from the brain The glymphatic system is more active during sleep, implying that astrocytes and AQP4-mediated water transport play a key role in sleep-related processes of waste clearance from the brain	Mouse	Iliff et al. [52]
Gliotransmission	In vitro optogenetic stimulation of astrocytic in the VLPO	Stimulation of astrocytes inhibits non-sleep-promoting neurons and excites sleep-promoting neurons TNAP is expressed in galanin-negative VLPO neurons, but not in galanin-positive sleep-promoting projection neurons	Rat	Choi et al. [76]
	In vivo optogenetic stimulation of VLPO astrocytes and pharmacology on cultured astrocytes	Stimulation of VLPO astrocytes increases the extracellular ATP concentration, c-Fos expression and sleep duration in the active phase in naturally sleep-waking animals Metabolic inhibition of VLPO astrocytes reduced ATP levels and sleep duration	Rat	Kim et al. [75]
	Enzymatic adenosine detection, Patch-clamp recordings, vascular reactivity	Differential sensitivity of astrocytes to glucose during the time-of-day. In the VLPO, glucose induces a threefold increase in adenosine release at the beginning of the resting period compared with its end	Mouse	Scharbarg et al. [7]
	Enzymatic quantification of adenosine, Immunohistolabelling and patch-clamp recordings	In the VLPO, astrocytes express the DP1 receptor for PGD_2_ Astrocytes induce adenosine release in response to PGD_2_ Astrocytes couple blood flow to neuronal activity	Mouse	Scharbarg et al. [103]
Metabolism	Fluorescence-activated cell sorting (FACS) and gene expression profiles	Sleep deprivation overexpresses genes encoding proteins involved in the astrocyte-neuron lactate shuttle (ANLS)	Mouse	Petit et al. [31]
Neurotransmitter recapture	Using a hypomorphic gat mutation (gat33-1)	A hypomorphic GAT mutation increased sleep amount, decreased sleep latency, and increased sleep consolidation at night	Drosophila melanogaster	Chaturvedi et al. [120]
Size of the astrocytic network/Synaptic Coverage/Metabolism	Conditional knockout of Cx43 in astrocytes, polysomnography, patch-clamp recordings, viral transduction, osmotic mini-pumps	Deletion of Cx43 exhibited increased sleepiness and fragmented wakefulness during their active (nocturnal) phase A reduced activity of orexin neurons due to impaired glucose and lactate trafficking through astrocytic networks	Mouse	Clasadonte et al. [36]
Transcriptional regulation	Translating ribosome affinity purification (TRAP), transcriptomic, ALDH1L1—eGFP-L10a BAC transgenic mice	Sleep/wake cycles regulate 1.4% of astrocytic transcripts linked to metabolism, extracellular matrix, and cytoskeleton Wakefulness brings astrocytic processes closer to synapses, and chronic sleep restriction increases astrocytic coverage	Mouse	Bellesi et al. [4]
Transcriptional Regulation/Metabolism	Two-Photon Imaging, Fluorescent dextrans and Radiolabeled compounds injections, FACS, Western Blotting, Electron Microscopy	• The mammalian brain-type fatty acid binding protein FABP7 which expression oscillates in tandem with the sleep–wake cycle regulates sleep consolidation across phylogeny FABP7 KO mice show fragmented sleep, similar to what is observed in human carriers of the FABP7 T61M mutation and transgenic flies carrying the murine Fabp7 or the Drosophila homolog dFabp	Human/Mice/Drosophila	Gerstner et al. [30]
Transcriptomic modulation	Cry1/2-null mice, Viral transfection, Calcium Imaging and locomotor activity recording	The astrocytic molecular clock alone is sufficient to orchestrate circadian oscillations within the SCN	Mouse	Brancaccio et al. [25]

Advances in transgenic animals, brain imaging, and molecular biology have permitted to deepen our understanding of astrocytes in sleep regulation. This review will explore how astrocytes integrate metabolism, circadian rhythms, and synaptic activity to regulate sleep at the molecular, cellular and network levels.

## Astrocytic gene expression: orchestrating circadian rhythms and sleep regulation

**Fig. 1:**
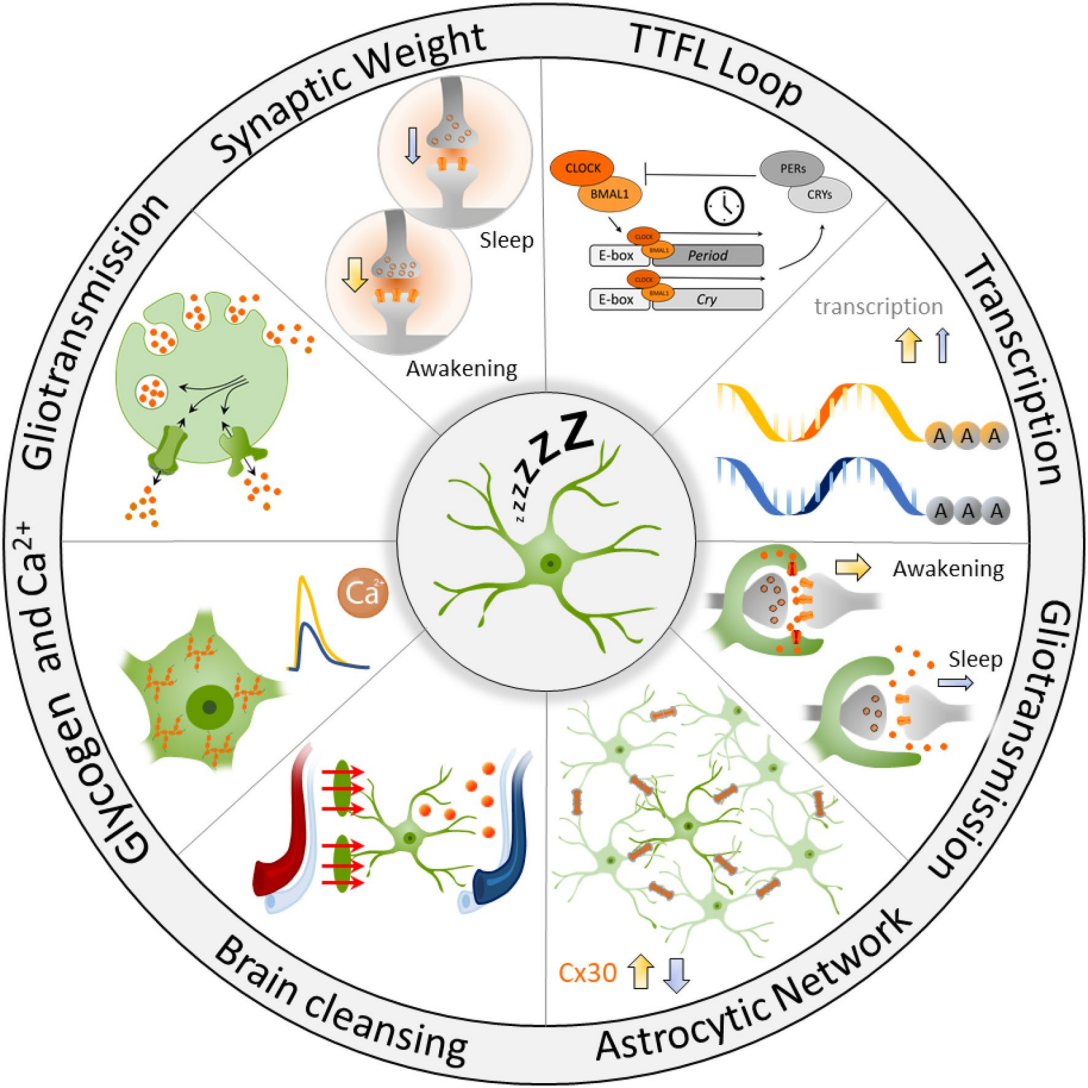
Schematic overview of the various mechanisms by which astrocytes regulate sleep. Astrocytes modulate circadian behaviors through their molecular clock, via a transcription-translation negative feedback loop (TTFL). They regulate gene transcription, thereby modulating synaptic coverage and the size of the astrocytic network. Variations in aquaporin-4 expression facilitate cerebrospinal fluid circulation and brain clearing. Astroglial calcium activity changes dynamically across vigilance states and contributes to sleep homeostasis. Intracellular glycogen storage and degradation by astrocytes contribute to the regulation of wakefulness and sleep. Astrocytes also participate in the reuptake of neurotransmitters and the release of gliotransmitters, which regulate the neuronal network. Additionally, astrocytes contribute to synaptic homeostasis by modulating synaptic weights, which may decrease their downscaling during non-rapid eye movement (NREM) sleep. Yellow arrows indicate wakefulness-related changes, while blue arrows represent sleep-related changes

Astrocytes are key players in the regulation of circadian rhythms, a fundamental sleep regulatory process. In the suprachiasmatic nucleus (SCN), transcriptional regulation has been observed in astrocytes, enabling a negative feedback loop (transcription-translation feedback loop, TTFL), and constituting an astrocytic circadian molecular clock (Fig. 1). In this loop, the expression of transcription factors, circadian locomotor output cycles kaput (CLOCK), and Brain and Muscle ARNT-Like protein (BMAL1), form a heterodimer in the nucleus to promote the expression of numerous genes containing the cis-acting E-box promoter element, including the period (*per*) and cryptochrome (*cry*) genes [24]. *Per* and *cry* proteins inhibit the CLOCK-BMAL1 complex, but they are destabilized by post-transcriptional regulations, such as phosphorylation/dephosphorylation and ubiquitination, which play a crucial role in regulating their expression during the circadian cycle. While TTFLs are not exclusive to astrocytes, as neurons also maintain autonomous circadian oscillations, recent research has demonstrated that this astrocytic clock alone is sufficient to drive circadian oscillations within the SCN and influence circadian behaviors in mice [25], highlighting the essential role of astrocytes in coordinating circadian physiology.

Moreover, the transcriptomic regulation of GABA transporters, specifically GAT3, facilitates the circadian rhythm of GABA uptake in the SCN. According to Patton et al. (2023), while GAT1 and GAT3 exhibit rhythmic expression, only GAT3 shows strong circadian regulation. GAT1 is expressed in both neurons and astrocytes, while GAT3 is predominantly astrocytic. Although both transporters contribute to GABA clearance, the authors demonstrated that this rhythmic GABA uptake is controlled by the cell autonomous circadian clock of astrocytes, with GAT3 playing a dominant role.

During the circadian day, the rhythmic upregulation of astrocytic GAT3 enhances GABA clearance, leading to lower extracellular GABA levels. This reduction in ambient GABA facilitates neuronal firing and promotes the release of neuropeptides essential for circadian signaling. In contrast, at night, the downregulation of GAT3 leads to an accumulation of extracellular GABA, which increases inhibitory tone and dampening neuronal excitability.

This circadian molecular clock within astrocytes orchestrates the day-night oscillation in GAT3 expression, driving its rhythmic transcription along with potentially other related genes. Through this time-of-day-dependent modulation of GABA uptake, astrocytes establish a dynamic environment that promotes neuronal activity during the day while exerting a more suppressive effect at night. This mechanism ensures the synchronization of GABAergic signaling with TTFL-driven circadian rhythms [26], emphasizing the integrative role of astrocytes in coordinating and sustaining circadian regulation, and the sleep–wake rhythm, at both the molecular and network levels (Fig. 1).

Transcriptional analysis of astrocytes from the cortex and striatum during the wake/sleep cycle has also revealed variations in gene expression throughout this cycle (Fig. 1). Using the technique of translating ribosome affinity purification (TRAP), which identifies mRNA undergoing translation, it has been shown that the astrocytic transcriptome changes between wakefulness and sleep, with 1.4% of astrocytic transcripts varying according to vigilance state [4]. Among these transcripts, 55 are overexpressed during sleep, such as *Cirp* and Uba1. *Cirp* encodes the cold-inducible RNA-binding protein, which binds to the untranslated regions of hundreds of transcripts to promote protein synthesis and cell proliferation and inhibits apoptosis [27], while *Uba1* encodes a ubiquitination-activating enzyme involved in protein degradation and synaptic homeostasis, both essential for proper sleep regulation. However, many more transcripts are overexpressed during wakefulness, 396 in total, particularly those involved in extracellular matrix formation and cytoskeletal protein synthesis, which are implicated in the extension of astrocytic processes [4]. Among these transcripts is the *gjb6* gene, which encodes connexin 30 (Cx30), a gap junction subunit controlling the morphological plasticity of astrocytes [28]. Astrocytes are indeed rapidly capable of modulating the extent of their peripheral astrocytic processes (PAPs), particularly in response to neuronal activity, which has been shown to directly influence synaptic glutamate levels in the hippocampus [28]. Thus, during wakefulness, astrocytic processes would be closer to synapses to facilitate glutamate clearance, whereas the reduction of astrocytic coverage during sleep could promote glutamate diffusion, to enhance neuronal synchronization, a hallmark of NREM sleep [4] (Fig. 1). Further experiments are required to determine whether these PAP movements effectively occur in relation to wakefulness and to explore whether such dynamics are consistent across distinct brain regions involved in regulating sleep and wakefulness.

Astrocytes also play a key role in sleep regulation through the circadian expression of mRNA encoding FABP7, a fatty acid-binding protein. As part of a large family of lipid chaperone proteins, FABP7 is essential in regulating intracellular fatty acid transport. Particularly enriched in astrocytes, FABP7 is involved in lipid signaling cascades that regulate changes in cellular growth, morphology and motility [29, 30]. The role of this protein in sleep regulation has been highlighted through the discovery of the T61M missense mutation in its gene, which leads to fragmented sleep in humans [30]. Similarly, its genetic deletion results in sleep fragmentation in mice [30].

Additionally, research into astrocytic gene expression during sleep has revealed an upregulation in the expression of the gene encoding the monocarboxylate transporter MCT1, which is essential for lactate transport between astrocytes and neurons [4]. This finding suggests an increased astrocyte-neuron lactate shuttle (ANLS) and restorative brain energy metabolism during sleep. Additionally, the increased glucose sensitivity observed in vitro, in astrocytes of the ventrolateral preoptic nucleus (VLPO), a key brain region involved in sleep regulation [7] further underscores the role of astrocytes in adjusting cerebral metabolism according to vigilance states. The influence of sleep deprivation on ANLS-related genes suggests that astrocytes dynamically adapt energy production and lactate transport to meet the fluctuating demands of wakefulness and sleep [31]. This ability of astrocytes to adapt metabolic processes is crucial for maintaining neuronal function and supporting sleep homeostasis, as the brain relies on these metabolic shifts for restoration and recovery during sleep.

## Morphological modulations of astrocytes: from synaptic coverage to astrocytic network

### Modulation of synaptic coverage

In the central nervous system, at least half of the synapses are enveloped by astrocytic processes known as perisynaptic astrocytic processes [12]. These fine processes are particularly enriched in ionic channels, neurotransmitter receptors, and transporters, allowing astrocytes to sense neuronal activity. Moreover, these extensions express proteins, such as ezrin, radixin, and connexin 30 (Cx30) [28, 32, 33], which contribute to the morphological plasticity of astrocytes. Through the non-channel functions of Cx30, changes in its expression levels can modulate intracellular cytoskeletal elements, leading to changes in astrocyte shape. Thus, the proximity of astroglial processes to the synapse permits the regulation of neurotransmission [28, 34]. Such changes in proximity to synapses have been observed by 3D electron microscopy during sleep regulation, as in the cortex, a few hours of sleep deprivation are sufficient to induce the extension of astrocytic processes closer to the synaptic cleft [4, 16] (Fig. 1).

### Modulation of the astrocytic network size

Sleep regulation may also involve the modulation of astrocytic coupling. Astrocytes are indeed interconnected via gap junctions, composed of Cx30 and 43 (Cx43). These gap junctions enable the direct intercellular exchange of small molecules between astrocytes, creating a functional network. The size of the network refers to the number of astrocytes that are connected and able to communicate via these junctions. Variations in connexin expression can expand or shrink the size of this network, which in turn influences neuronal excitability [17] and consequently, sleep regulation [35].

The conditional *Cx43* deficiency in astrocytes results in nocturnal hypersomnolence [36]. Modafinil, a potent wake-promoting psychostimulant, selectively upregulates Cx30 expression in the cortex without affecting Cx43 levels, potentially enhancing astrocytic coupling [37]. In contract, sleep-promoting agents such as gamma-hydroxybutyric acid or oleamide reduce astrocytic coupling, reinforcing the notion that astrocytic network connectivity is dynamically regulated across the sleep–wake cycle [38, 39]. These findings suggest that astrocytic coupling increases during wakefulness and decreases during sleep. Astrocytic networks play a pivotal role in regulating neuronal activity and sleep processes, dynamically adapting their size in response to physiological demands. This dynamic modulation may either mirror the activity levels of the underlying neuronal networks or exert direct influence over neuronal circuits. For example, when neuronal activity is suppressed using tetrodotoxin (TTX), astrocytic network size decreases significantly [20]. Conversely, heightened activity in the visual cortex, such as that induced by visual experience, induces a robust increase in Cx30 expression by approximately 70% [40].

Importantly, experimental manipulations of Cx30 expression further underscore its regulatory role. In Cx30-deficient mice, both astrocytic network size and neuronal activity are decreased [28, 41]. Conversely, viral overexpression of Cx30 expands the astrocytic network size but, paradoxically, also reduces neuronal activity [17]. These findings indicate that astrocytes finely tune neuronal excitability by modulating their network size, positioning them as dynamic regulators of excitability and sleep–wake transitions.

The evidence supports the idea that astrocytic networks are physiologically optimized to balance neuronal excitability and facilitate processes such as sleep regulation. A disruption in this optimization, either through reduced or excessive network size, can potentially impair neuronal function and sleep dynamics, underscoring the critical interplay between astrocytes and neurons in maintaining brain homeostasis.

## Astrocytic roles in metabolite clearance and glycogen storage

### Metabolite clearance in the brain

For over a decade, an interesting observation has been made regarding the importance of sleep for brain metabolite clearance. In vivo microdialysis experiments have shown that beta-amyloid peptide (Aβ) levels in the central nervous system’s interstitial fluid are higher during wakefulness than during sleep [42], with these variations decreasing as Alzheimer's disease progresses [43]. It has been proposed that the glymphatic drainage system may allow for the elimination of this Aβ peptide and that dysfunction of this drainage during sleep may contribute to the progression of the disease [44]. At the cortical level, sleep is associated with a ~ 60% increase in the interstitial space, which facilitates metabolite clearance [45, 46] (Table 1 and Fig. 1). However, these results remain debated [48], as Miao et al., suggest the opposite, showing that brain clearance may decrease during sleep due to limited bulk flow and diffusion dynamics [47]. These contrasting observations may stem from methodological differences among studies, particularly regarding the injection site of tracers, into the cerebrospinal fluid or directly into the brain parenchyma, as well as potential alterations in intracranial pressure, due to differences in infusion rates, injection volumes, or anesthesia protocols (e.g., isoflurane vs. ketamine-xylazine). These factors can differently influence cerebrospinal fluid dynamics and clearance efficiency by modulating cerebrovascular tone, glymphatic flow, and neuronal activity. Furthermore, the physicochemical properties of the injected molecules, including their molecular weight, charge, solubility, and affinity for specific transporters, may differentially affect their diffusion, uptake, and elimination across brain compartments. These different results highlight the need for standardized methodologies in tracer infusion and imaging techniques, methodology using non-invasive approaches and further investigations into the influence of regional heterogeneity on glymphatic clearance.

The primary mechanism responsible for this drainage involves aquaporin-4 (AQP4), a water-permeable channel essential for fluid transport. AQP4, one of the 13 members of the aquaporin family [48], is predominantly expressed by astrocytes at perivascular endfeet in contact with blood vessels [49]. Astrocytes may function as valves, actively promoting cerebrospinal fluid (CSF) flow through perivascular spaces, while preventing backflow [50, 51]. Deletion of AQP4 disrupts glymphatic clearance [52], underscoring the critical role of astrocytes in waste removal during sleep (Fig. 1).

Recent research provides further insights into the mechanisms driving glymphatic clearance. Nedergaard’s group demonstrated that in vivo, cerebrospinal fluid movement is facilitated by rhythmic blood vessel contractions regulated by norepinephrine oscillations, particularly during NREM sleep [53]. These oscillations create a pumping mechanism, propelling cerebrospinal fluid deeper into the brain, thereby enhancing waste clearance. Importantly, the study found that the widely used sleep aid zolpidem (Ambien) disrupts these blood vessel oscillations, potentially impairing glymphatic function and raising concerns about its impact on neurodegenerative diseases [53]. In addition to waste removal, astrocytes play a key role in brain metabolism by dynamically storing and utilizing glycogen to support neuronal function.

### Glycogen storage for sleep and wakefulness consolidation

Glycogen is primarily stored in astrocytes and serves as an energy reservoir, breaking down into glucose and lactate in response to increased neuronal activity, thereby fueling neuronal metabolism [54]. While direct neuronal glucose uptake [55], mitochondrial function [56], and vascular supply [57] also play essential roles in sustaining energy homeostasis, astrocytes have been identified as key metabolic hubs. Heller and Benington (1995) were the first to hypothesize that the restoration of astrocytic glycogen during NREM sleep is a crucial function of this state [58], counteracting the depletion of cerebral glycogen stores that occurs during wakefulness [59, 60]. However, the observation of increased brain glycogen after sleep deprivation led to the "glycogenetic" hypothesis, which proposes that both glycogen synthesis and utilization increase during wakefulness. At the same time, reduced excitatory transmission during sleep creates an imbalance that causes glycogen accumulation [61]. This suggests that glycogen renewal primarily occurs during wakefulness [62]. Notably, overexpression of glycogen synthase kinase-3 β, a key enzyme in glycogen synthesis results in fragmented wake-sleep states, without affecting the total amount of sleep or wakefulness [63]. Therefore, rather than merely replenishing cellular energy stores, astrocytic glycogen storage and degradation appear to be crucial for maintaining both wakefulness and sleep cycles (Fig. 1). Beyond energy storage, astrocytes also exhibit intracellular dynamic calcium fluctuations, which are essential for modulating neuronal activity, gliotransmission, and sleep–wake transitions.

## Calcium signaling in astrocytes and its influence on sleep–wake regulation

### Physiological astrocytic Ca^2+^ fluctuations during the sleep–wake cycle

Growing evidence suggests that intracellular calcium (Ca2 +) signaling in astrocytes play a key role in sleep homeostasis. Recent in vivo imaging studies demonstrated that cortical astroglial Ca^2^⁺ levels fluctuate across vigilance states, reaching higher levels during wake and lower levels during NREM and REM sleep, with these changes predominantly occurring in astrocytic processes rather than somata [64]. These changes do not appear to simply reflect passive responses to surrounding neuronal activity, as cortical astrocytic Ca^2+^ displays distinct state-dependent patterns: synchronized activity that peaks during wakefulness [64], while cortical neurons reach peak synchronization during the slow-wave oscillations of deep sleep [65, 66]. Additionally, astrocytic Ca^2+^ activity exhibits dynamic shits that often precedes changes in neuronal firing patterns, suggesting a proactive role in driving transitions between sleep states [67, 68]. At the cortical level, the frequency of astrocytic Ca^2+^ events indeed increases just before the onset of slow-wave oscillations, characteristics of deep NREM sleep, a pattern that persists throughout sleep cycles [66]. Notably, further increases in astrocytic Ca^2^⁺ event frequency occur immediately before the transition into rapid eye movement (REM) sleep, and before awakening [70, 71]. Moreover, individual Ca^2+^ events in cortical astrocytes tend to be longer during wakefulness than sleep, with both the amplitude and duration of these events varying across wakes and sleep states [69]. Together, these findings suggest that astrocytes not only participate in, but also play an active role in modulating the neural processes underlying sleep–wake transitions and cortical synchrony (Table 1).

The essential role of astrocytic calcium signaling in sleep regulation has also been highlighted by the genetic ablation of the inositol 1,4,5-trisphosphate receptor 2 (*IP*_*3*_*R2*), a receptor almost exclusively expressed in astrocytes. IP3R2 activation leads to the release of Ca^2+^ from intracellular stores, particularly the endoplasmic reticulum and at the somatic level and major branches [70]. Its deletion selectively disrupts astrocytic Ca^2+^ signaling, leading to fragmented NREM sleep and a decrease in delta power during NREM sleep [67]. The involvement of astroglial Ca^2+^ has also been investigated by suppressing the transmembrane protein STIM1 (Stromal Interaction Molecule 1), which acts as a calcium sensor in the endoplasmic reticulum. When Ca^2+^ levels decrease in the endoplasmic reticulum, STIM1 is activated and initiates Ca^2+^ entry through the stores (Store-Operated Calcium Entry, SOCE). Thus, the specific suppression of STIM1 in astrocytes leads to a decrease in NREM sleep activity after sleep deprivation. These results suggest that cortical astrocytic Ca^2+^ contributes to sleep homeostasis [64].

All these findings underscore the crucial role of astrocytic Ca^2+^ signaling in sleep regulation (Fig. 1). However, it is noteworthy that these Ca^2+^ dynamics exhibit regional variability in the brain, particularly during NREM sleep [68, 71, 72]. During REM sleep, astrocyte Ca^2+^ levels consistently decrease across areas such as the cortex, hippocampus, hypothalamus, and brainstem. Upon waking, a rapid increase in Ca^2+^ levels occurs. In contrast, during NREM sleep, the changes in Ca^2+^ levels are more region-specific. In the cortex and hippocampus, Ca^2+^ levels significantly decrease, whereas in the hypothalamus and brainstem, these levels remain stable compared to wakefulness [68, 71, 72]. Therefore, the physiological role of astrocytes in sleep–wake regulation appears to be brain region-dependent.

### Astrocytic Ca^2+^ variations induced by optogenetic or DREADD stimulations and effects on vigilance states

Recent studies using optogenetic or chemogenetic approaches provided further compelling evidence for the involvement of astrocytes in sleep regulation, revealing region-specific effects. However, while these findings are intriguing, they also raise significant methodological and interpretative challenges.

For instance, optogenetic stimulation of astrocytes in the posterior hypothalamus, a region involved in both wakefulness and sleep, transiently increases both NREM and REM sleep duration during circadian periods when rodents are typically awake [73]. Interestingly, this sleep induction did not enhance slow-wave activity, suggesting that astrocytes in this region primarily influence sleep timing and consolidation, rather than the intensity of slow-wave oscillations [73].

In contrast, chemogenetic activation of hippocampal astrocytes reduced the total time in wakefulness, while increasing the total sleep time, without affecting cortical oscillations across sleep–wakefulness states [74]. Conversely, astrocytic stimulation in the pons selectively impairs REM sleep, delaying REM onset and reducing the number of REM episodes. Interestingly, this regional modulation was accompanied by an enhancement of delta wave activity during NREM sleep [74].

Further supporting this regional specificity, targeted optogenetic stimulation of astrocytes in the VLPO, a key center for regulating NREM sleep, increases sleep duration during the active phase of mice [75]. Experiments in brain slices indicate that this astrocytic stimulation in the VLPO can inhibit local interneurons, while simultaneously depolarizing sleep-promoting neurons, suggesting that this astrocytic stimulation may induce the release of a signaling molecule with dual excitatory and inhibitory effects, such as adenosine, to promote sleep [76]. However, it remains unclear whether astrocytic adenosine release follows a physiologically relevant time course or is an artifact of artificial activation.

To further elucidate the role of astrocytic calcium activity, studies of astrocytic calcium dynamics in the cortex revealed that calcium transients in astrocytes precede spontaneous shifts to slow-oscillation states, characteristic of NREM sleep. Moreover, optogenetic activation of cortical astrocytes can trigger these states by altering extracellular glutamate levels [66]. Using two-photon glutamate imaging, studies have also demonstrated under anesthesia that astrocyte-evoked extracellular glutamate spikes coincide with neuronal circuit synchronization [66]. Remarkably, single electrophysiological stimulation of an astrocyte under anesthesia is sufficient to activate nearby astrocytes and induce membrane fluctuations in adjacent neurons, leading to network-driven "UP states", as visualized by two-photon imaging [77].

These findings collectively suggest that astrocytes play an active role in controlling cortical synchronizations, primarily through the modulation of extracellular glutamate, thereby stabilizing neuronal dynamics during sleep [66, 78]. Astrocytes appear capable of enhancing local synchrony without directly increasing cortical excitability to levels associated with wakefulness [77]. During NREM sleep, although cortical astrocytic Ca^2^⁺ levels are typically low [67], astrocytes nonetheless contribute meaningfully to the oscillatory and highly synchronized states that define this phase [66, 79]. Rather than driving activation, astrocytes support and reinforce the stability of these synchronized networks [77]. Conversely, during wakefulness, cortical astrocytes exhibit high Ca^2^⁺ levels [64] that are associated with increased extracellular glutamate [66], which promotes neuronal desynchronization, a hallmark of the awake state. This high astrocytic Ca^2^⁺ activity plays a pivotal role in the regulation of arousal, as it actively supports the dynamic and desynchronized neuronal environment needed for alertness and cognitive processing.

However, interpreting results from optogenetics and chemogenetics studies requires caution, as the frequencies, intensities, and stimulation patterns used in such experiments may exceed physiological levels. Moreover, astrocytes are highly polarized cells, and the abrupt, widespread opening of rhodopsin channels or activation of chemogenetic constructs can represent an artificial activation profile that might not accurately reflect astrocytic signaling.

Activation of cortical astrocytes, artificially and selectively expressing receptors solely activated by synthetic drugs (DREADD), has shown that different astroglial Ca^2+^ signaling pathways, although convergent, distinctly modulate sleep components. It has thus been demonstrated that cortical activation of Gi-DREADD, which increases the frequency of Ca^2+^ events, induces an increase in slow-wave activity (SWA) during slow-wave sleep without influencing its duration. Conversely, activation of Gq-DREADD in these astrocytes prolongs the duration of NREM sleep without influencing SWA [68]. Finally, Gq-DREADD activation of basal forebrain astrocytes induces prolonged and uninterrupted periods of wakefulness, without a homeostatic response (increase in sleep duration or intensity), typically observed following sleep deprivation [80].

Altogether, these results highlight the essential role of astrocytic Ca2 + signaling in sleep regulation, not merely as a passive reflection of neuronal activity. However, their precise role appears to be complex and context-dependent. While astrocytic Ca^2^⁺ activity appears to shape neuronal excitability and network dynamics, whether this occurs primarily through gliotransmission, metabolic support, or modulation of extracellular ionic balance remains an open question. Additionally, the interplay between Ca^2^⁺ transients and overall Ca^2^⁺ levels varies regionally, reinforcing the need for spatiotemporal specificity in future investigations. Moreover, the need for sleep may depend not only on how long an individual has been awake, but also on specific patterns of activity within both neuronal and glial circuits. Beyond their role in sleep–wake transitions, astrocytic Ca^2^⁺ dynamics influence gliotransmitter release, thereby modulating synaptic communication and network synchronization, further reinforcing their role in sleep homeostasis.

## Astrocytic modulation of sleep through the release of gliotransmitters and sleep-regulating substances

Gliotransmission, described as the "active transfer of information from glia to neurons", is a complex phenomenon that encompasses various types of signaling and released molecules [81]. Many studies have reported that the release of gliotransmitters occurs via Ca^2+^-dependent exocytosis of vesicles [82–84] and lysosomes [85, 86]. Astrocytes indeed possess all the molecular machinery necessary for exocytosis, including proteins of the soluble N-ethylmaleimide-sensitive factor attachment protein receptor (SNARE) complex [87, 88], vesicular pumps and transporters [89, 90]. Furthermore, studies of astrocyte ultrastructure have revealed the presence of vesicles of various apparent sizes ranging from ~ 100 to 600 nm in diameter [91], as well as the localization of vesicles approximately thirty nanometers in diameter close to synaptic terminals [82]. Other modes of astroglial release not dependent on Ca^2+^ have also been described, involving the reversal of neurotransmitter transporters' polarity or the opening of hemichannels such as connexins [35] and pannexins [92]. However, the physiological and/or pathological conditions under which these different mechanisms occur remain unclear.

### Adenosine

Astrocytes can release numerous molecules, some of which influence the regulation of sleep (Fig. 1). Adenosine release has certainly been the most studied, given the central role of this purine in the homeostatic regulation of sleep [93]. The accumulation of adenosine in the extracellular space is thought to result from the high ATP consumption during wakefulness [94, 95], particularly in brain regions such as the cortex and hippocampus [94, 95], as well as the hypothalamus [7]. This accumulation is believed to encode the need for sleep [58, 96].

Astrocytic exocytosis has long been widely recognized as a key mechanism involved in the release of adenosine by astrocytes, particularly in the regulation of sleep homeostasis. Early evidence supporting this hypothesis comes from studies using mutant mice expressing a dominant-negative form of the SNARE complex in astrocytes (dnSNARE), in which “astrocyte-specific” expression was shown to impair sleep homeostasis after sleep deprivation, of both NREM sleep and REM sleep duration, while control mice exhibited increased duration of sleep and episodes of deep NREM sleep following sleep deprivation [97]. However, subsequent studies have challenged the specificity of this model, based on the GFAP promoter used in these SNARE mice, which was found to leaky expression in neurons, that could confound interpretations of astrocyte-derived adenosine signaling [98]. Furthermore, direct measurements of extracellular adenosine levels using adenosine sensors of the G protein-coupled receptor activation-based type (GRAB-Ado), raised questions about whether astrocytes are the primary source of adenosine in sleep regulation. It has indeed been shown that in the basal forebrain, neurons, rather than astrocytes, represent an important source of adenosine [99]. Although the level of Ca^2+^ in astrocytes is positively correlated with the amount of extracellular adenosine, experimental suppression of astrocytic Ca^2^⁺ transients had no effect on adenosine levels in the basal forebrain [100]. This suggests that astrocytes may influence adenosine metabolism or transport, but their direct contribution to extracellular adenosine release remains unclear.

Additionally, adenosine production via ATP release from astrocytes may occur independently of Ca^2^-mediated exocytosis, as *IP3R2*-KO mice still exhibit Ca^2+^ transients in astrocytic processes, which may be crucial for adenosine production. Supporting this, optogenetic stimulation of VLPO astrocytes, known to increase sleep duration, has been shown to trigger ATP release by hemichannels and/or P2X7 receptors [75]. This ATP is subsequently degraded into adenosine by local interneurons through the activation of the Tissue-nonspecific Alkaline Phosphatase (TNAP) enzyme [76] (Table 1).

Taken together, these findings suggest that while astrocytes may play a role in adenosine homeostasis, their function as a primary source of adenosine release remains debated. The discrepancy between dnSNARE-based studies and direct adenosine measurements highlights the need for more refined transgenic models with improved cell-type specificity. Future research should aim to clarify the regional specificity of astrocytic ATP-to-adenosine conversion, particularly in sleep-regulating areas such as the VLPO and basal forebrain. It is also essential to distinguish between direct astrocytic adenosine release and the modulation of extracellular adenosine levels through ATP hydrolysis. Additionally, advancing high-resolution, real-time imaging techniques will be crucial for tracking adenosine dynamics in vivo across different sleep–wake states.

**Fig. 2:**
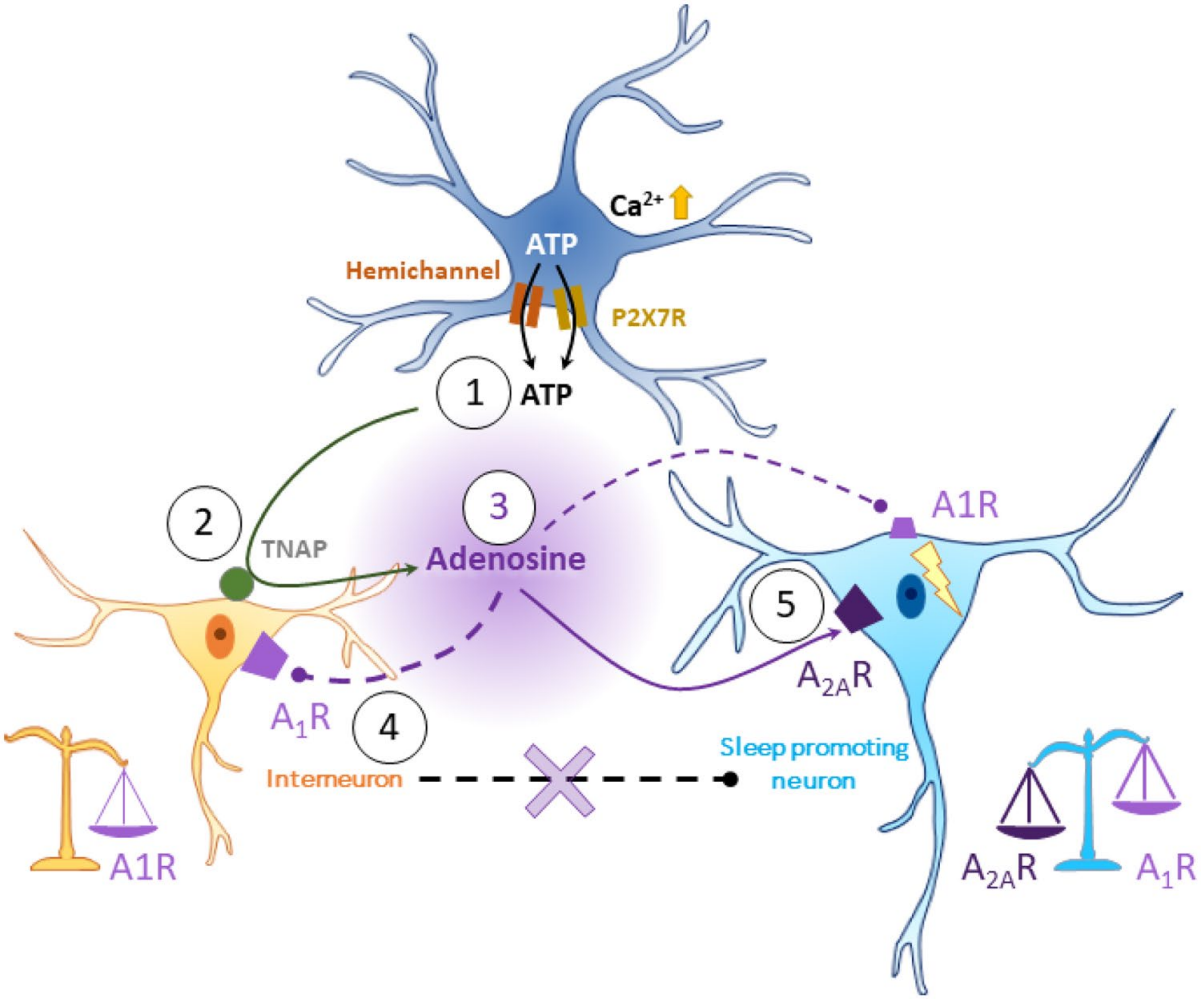
Schematic representation of adenosine origin and mode of action in the VLPO. Following astrocytic activation, intracellular Ca2 + levels increase, leading to the release of ATP via hemichannels or P2X7 receptors (1). This ATP is then cleaved into adenosine by tissue-nonspecific alkaline phosphatase (TNAP) enzymes (2) located on local interneurons. The adenosine (3) acts on A_1_R on these interneurons, reducing their inhibitory action on sleep-promoting neurons (4). Simultaneously, adenosine inhibits sleep-promoting neurons through A_1_R and activates them via A_2A_R, which are more highly expressed

In vitro enzymatic measurements of adenosine levels further indicate that adenosine, once released into the extracellular space, also accumulates in the VLPO during wakefulness to exert sleep pressure [7]. Furthermore, astrocytes exhibit a time-depend regulation of their response to glucose and adenosine production, acting as temporal integrators. Thus, the same variation in glucose concentration in the VLPO induces a threefold increase in adenosine release at the beginning of the rest period compared to the end [7]. Adenosine promotes sleep by modulating neuronal excitability across multiple brain regions via A_1_ and A_2A_ adenosine receptors (A_1_R and A_2A_R), which exert inhibitory and excitatory effects, respectively (for review, see [101]). In the VLPO, in vitro studies have shown that adenosine indirectly disinhibits sleep-promoting neurons by inhibiting local interneurons [7, 102], while also directly stimulating sleep-promoting neurons via A_2A_ receptor activation [76, 103]. However, since sleep-promoting neurons also express A_1_ receptors, as local interneurons [7, 104], specific injection of adenosine A1R agonists into the VLPO increases wakefulness [105] (Fig. 2). Notably, the A_2A_R/A_1_R expression ratio on sleep-promoting neurons appears to be species-dependent. In rats, adenosine application inhibits sleep-promoting neurons [104], whereas in mice, it induces depolarization [7] (Table 1), emphasizing the need for species-specific investigation into adenosinergic regulation of sleep.

### Prostaglandin D_2_

Astrocytes play a key role in sleep regulation through the release of prostaglandins, notably prostaglandin D_2_ (PGD_2_) [106] and prostaglandin E2 [107]. These releases are regulated by Ca^2+^ oscillations within astrocytes [108]. Among these molecules, PGD_2_ is one of the most potent endogenous sleep-promoting molecules [103], with its concentration in the cerebrospinal fluid fluctuating according to the circadian rhythm of wakefulness and sleep [109, 110]. Astrocytes also express the DP1 receptor for PGD_2_, enabling them to respond to PGD_2_ activation. This interaction enhances extracellular adenosine concentrations, thereby simultaneously adjusting blood flow and neuronal activity within the VLPO [103]. Through these mechanisms, astrocytes effectively integrate metabolic and neuronal signals to regulate sleep–wake dynamics.

### Lactate

Astrocytes can release lactate, the extracellular variations of which fluctuate during wake-sleep cycles [63, 108]. A mechanism known as the astrocyte-neuron lactate shuttle (ANLS) has been proposed to play a fundamental role in sleep regulation by linking neuronal activity to energy supply [31]. According to this mechanism, astrocytes take up glutamate released during neuronal activity and convert it into lactate for subsequent release, directly linking neuronal activity to lactate energy supply by astrocytes. Neurons are, however, directly sensitive to glucose [55] and may even prefer glucose over lactate as the primary energy source during periods of sustained activity [111]. Nevertheless, beyond the metabolic role of lactate, its release by astrocytes could directly contribute to the slowing of cortical neuron discharge while falling asleep [112]. Another effect of lactate on neurons has been reported by Yang and colleagues, who showed that genes related to neuronal plasticity such as Arc and Zif268 were stimulated in vitro and in vivo by lactate application [113]. This increase is thought to be mediated by NMDA receptor activation and the Erk1/2 signaling pathway [113]. Unlike the direct electrophysiological effects of lactate, lactate transport via monocarboxylate transporters (MCTs) is necessary to exert its effects [114]. These results indicate that, at the level of a single synapse, lactate could simultaneously support synaptic activity as an energy substrate and directly participate in activity-related plasticity mechanisms by triggering the transcription of plasticity-related genes. These lactate effects could contribute to memory mechanisms associated with sleep.

### Neurotransmitters

Astrocytes can release glutamate via exocytosis, a process dependent on Ca^2+^ [3, 71], which involves a specific subpopulation of astrocytes expressing the vesicular glutamate transporter VGLUTs [115]. This release can also be modulated by changes in cell volume, thereby activating glutamate-permeable channels [116]. Moreover, exocytosis in astrocytes is stimulated by morphological changes induced by cyclic AMP, leading to increased glutamate release [117]. *Ex-vivo* experiments on brain slices demonstrated that this astrocyte-derived glutamate release promotes neuronal synchronization, a hallmark of NREM sleep [66].

Astrocytes also play a role in sleep physiology, particularly through the regulation of GABAergic signaling. They actively participate in the uptake of GABA, a neurotransmitter that plays a key role in sleep regulation [118]. In mammals, GABAergic neurons, within key sleep-regulating regions such as the median preoptic nucleus (MnPO), VLPO nuclei, suprachiasmatic nucleus (SCN), and parafacial zone [119], play a crucial role in the induction and maintenance of NREM sleep. The GABAergic nature of these neurons underscores the importance of astrocytic GABA uptake in fine-tuning sleep architecture. Astrocytes influence sleep onset and duration by modulating extracellular GABA levels. Pharmacological inhibition of the GABA transporter-1 (GAT-1), a key astrocytic transporter, shortens the latency to NREM sleep onset while simultaneously increasing the duration of NREM sleep and the number of sleep episodes [118]. These findings suggest that astrocytes regulate the availability of GABA at synapses, ensuring proper inhibitory tone necessary for sleep initiation and maintenance.

Similarly, in Drosophila, astrocytic regulation of extracellular GABA levels has emerged as a key mechanism in sleep regulation. The hypomorphic mutation *gat* (gat33-1) in the GAT-1 gene, encoding the astrocytic GABA transporter, leads to increased sleep duration and consolidation while reducing the latency to sleep onset [120]. These effects are attributed to an increase in extracellular GABA, which dampens the activity of wake-promoting circadian neurons, further reinforcing the conserved role of astrocytic GABAergic signaling in sleep regulation across species.

Together, these findings highlight the essential contribution of astrocytes to sleep homeostasis via GABAergic modulation. By controlling extracellular GABA levels, astrocytes not only regulate the balance between wakefulness and sleep, but also influence the stability and consolidation of sleep states. This underscores the need for further research into astrocytic GABA dynamics, particularly in different brain regions, to fully understand their region-specific contributions to sleep physiology.

### Cytokines

Astrocytes play a significant role in regulating sleep, in part through the release of cytokines, such as interleukin 1 (IL-1). While astrocytes are a source of IL-1β, this cytokine is not exclusively produced by astrocytes; it is also expressed in microglia and, to a lesser extent, neurons [121]. Studies have shown that IL-1β mRNA levels exhibit diurnal fluctuations in sleep-regulatory brain regions. In the hypothalamus, hippocampus, and cortex, IL-1β mRNA levels peak shortly after the lights-on, decline during the reminder of the light period, and stay low during the dark phase [122]. In contrast, no significant changes in IL-1β mRNA levels were observed in the brainstem or cerebellum. These regional variations in IL-1β mRNA expression suggest a region-specific role for IL-1β in sleep–wake regulation. The cellular origin of these IL-1β mRNA fluctuations remains unclear, as both astrocytes and neurons express IL-1β mRNA in the brain. However, astrocytes are thought to be key contributors to IL-1β-mediated sleep regulation. Indeed, when IL-1β derived from cultured astrocytes are injected into the ventricles of rats, there is an increase in slow-wave sleep activity [123]. Similarly, the inhibition of IL-1 and TNF-α via antibodies or antagonists has been found to reduce slow-wave sleep, whereas their administration increases it [123, 124]. Recent in vivo research provides deeper insight into these processes. In transgenic mice that express IL-1 receptor 1 (IL1R1) selectively in the central nervous system and either on neurons or astrocytes, it was shown that in mice with IL-1 receptor 1 (IL1R1) expressed on astrocytes exhibit greater sleep fragmentation and delayed increases in NREM delta power compared to those with IL1R1 expressed on neurons [125]. Additionally, research in fruit flies demonstrates that calcium signaling in astrocytes can promote sleep by upregulating the expression of the monoamine receptor TyrRII and triggering the release of Spätzle, an IL-1 analog, which activates sleep-promoting neurons [126]. Together, these findings underscore a crucial, cell-specific role for astrocyte-derived IL-1 signaling in sleep regulation, further linking immune responses to sleep modulation in both mammals and invertebrates.

### BDNF

The case of Brain-Derived Neurotrophic Factor (BDNF) is interesting because it is primarily synthesized by neurons, then internalized, matured, and finally released by astrocytes [127]. In addition to a role in cell growth and synaptic long-term potentiation (LTP), BDNF appears to be involved in sleep homeostasis. Studies have shown in mice that intracortical injection of BDNF at the beginning of the wake period increases the intensity of slow-wave sleep, measured by slow-wave activity (SWA) [128]. Conversely, in transgenic rats with a 50% reduction in BDNF levels, the duration of slow-wave sleep episodes is decreased [129]. Loss of BDNF expression dependent on the BDNF promoter IV has also been associated with decreased SWA during slow-wave sleep, suggesting altered sleep homeostasis [130].

BDNF exerts its effects via its receptor, tropomyosin-related kinase B (TrkB), which exists in two main isoforms: TrkB.FL, predominantly expressed by neurons, and TrkB.T1, the truncated isoform predominantly found in astrocytes [131]. TrkB.T1 lacks the intracellular tyrosine kinase domain. To explore the specific role of astrocytes and the TrkB.T1 receptor in sleep, researchers examined sleep patterns in mice genetically modified to lack TrkB.T1. These TrkB.T1-null mice exhibit normal NREM sleep time and NREM sleep homeostasis [132]. However, they display several alterations commonly observed in mood disorders. These include increased REM sleep time, reduced REM sleep latency, and sleep fragmentation, characterized by shorter bouts of both sleep and wakefulness episodes similar to those seen in major depressive disorders [132].

These results suggest that astrocytic TrkB.T1 plays a role in maintaining sleep integrity and may provide a molecular link between sleep abnormalities and psychiatric conditions. As astrocytes are also known to release a variety of other gliotransmitters in response to local brain activity [133], the full spectrum of their influence on sleep remains to be fully elucidated. This growing body of evidence underscores the importance of astrocyte-mediated BDNF signaling in sleep regulation and the broader context of brain health.

## Synaptic regulation by astrocytes: pruning, plasticity, and sleep-associated downscaling

Astrocytes play a crucial role in maintaining synaptic homeostasis in the adult brain by regulating synaptic pruning through phagocytosis. In the hippocampus, astrocytes eliminate unnecessary excitatory synapses via the phagocytic receptor MEGF10, while inhibitory synapses are spared [134]. In the cerebellum, Bergmann glial cells utilize ABCA1, another phagocytic receptor, to regulate synapse size rather than reduce synapse numbers [135]. Additionally, in the cerebral cortex, astrocytes are involved in the elimination of presynaptic elements, especially under conditions like sleep deprivation, where increased astrocytic expression of the receptor MERTK is observed and increased lipid peroxidation in synaptoneurosomes [16]. These findings suggest that astrocytic phagocytosis may represent the brain’s adaptive response to the heightened synaptic activity during prolonged wakefulness. This process likely facilitates the maintenance of neural homeostasis by clearing worn-out components from heavily utilized synapses. Future research is needed to determine how astrocytic receptors MEGF10, ABCA1, and MERTK differentially recognize and control the pruning of different synapse types across various neural circuits [135–137].

The interactions between astrocytes and neurons also enable astrocytes to play a role in regulating long-term synaptic plasticity (LTP and LTD) [17, 138]. Their ability to form a more or less extensive astrocytic network, along with the regulation of the proximity of their processes to synapses, modulates the volume of the extracellular space and their capacity to regulate extracellular levels of glutamate and potassium. It has also been hypothesized that TNFα release by astrocytes could mediate the signaling pathways involved in sleep-associated synaptic weakening [139]. However, since the in vitro effects of TNFα on glutamatergic and GABAergic synapses are diverse [140, 141], it remains unclear whether glial-derived TNFα signaling provides a plausible molecular mechanism for synaptic weakening during sleep.

During deep slow-wave sleep, a crucial process of pruning and reducing synaptic connections in the brain has been hypothesized to be essential for cognitive health and memory. The Synaptic Homeostasis Hypothesis (SHY), primarily formulated by Giulio Tononi and Chiara Cirelli, suggests that deep slow-wave sleep plays a regulatory role in adjusting cerebral synaptic weights [142]. According to this hypothesis, during wakefulness, synapses are strengthened by learning and experiences, a fundamental process for memory formation. However, continuous increases in synaptic activity, without regulation, could lead to synaptic saturation and increased energy expenditure. Sleep, particularly during deep slow-wave sleep, may then serve as a "reset" for synapses, reducing their activity through a process of "downscaling" (Fig. 1). The SHY hypothesis predicts that this downscaling is not uniform for all synapses, but that some, those associated with "memories", are protected and remain stable during sleep [143]. This mechanism preserves essential information acquired throughout the day while eliminating excess connections, thus enabling new learning possibility every day. In a way, sleep is the price we pay for learning and memory formation. However, while it has been well established that astrocytes play a crucial role in modulating synaptic activity and plasticity, neurons also actively participate in this process through synaptic tagging, and activity-dependent modifications. Their coordinated interactions with astrocytes ensure appropriate synaptic downscaling and memory consolidation. However, the precise molecular interplay between astrocytic and neuronal contributions to synaptic weakening during sleep—essential for cognitive function, memory consolidation, and overall brain health—remains an open question that requires further investigation.

## Conclusion

Long underestimated, the role of astrocytes in sleep regulation now appears to be undeniable. These cells are dynamic elements, fully equipped to integrate local information. Astrocytes act as integrators of energy metabolism, sleep pressure, and circadian timing to appropriately regulate and coordinate neuronal activities, neuronal networks, and sleep thanks to gene transcription, Ca^2+^ signaling pathways, their function as energy reservoirs, synaptic coverage plasticity, gliotransmission, and their ability to regulate long-term synaptic efficiency.

### Developmental dynamics and astrocytic heterogeneity

The role of astrocytes evolves throughout life, encompassing various functions from development to senescence [144, 145]. During these extreme periods of life, both astrocytes function and sleep architecture undergo significant changes, highlighting the need for further research into how astrocytes contribute to sleep regulation over time. Moreover, astrocytes are not a uniform population. They exhibit marked heterogeneity across brain regions and subtypes, reflecting not only developmental origins, but also regional specialization and evolutionary influences [146]. This diversity likely translates into region-specific contributions to sleep regulation, as astrocytes in different brain areas express distinct receptors, as well as the brain regions where they are located.

For instance, cortical astrocytes have been shown to modulate slow-wave activity (SWA) during NREM sleep by regulating extracellular glutamate levels and promoting neuronal synchronization [66]. Conversely, astrocytes in the basal forebrain appear to be critical for wakefulness maintenance, as their stimulation leads to prolonged arousal without triggering compensatory sleep rebound [69]. Similarly, astrocytes in the VLPO, are thought to enhance NREM sleep through adenosine release, which exerts differential effects on wake-active and sleep-promoting neurons [76, 103].

Beyond these region-specific functions, astrocytes also play a role in sleep homeostasis, with their intracellular Ca^2^⁺ dynamics encoding sleep need. Studies have demonstrated that astrocytic Ca^2^⁺ activity is highest during wakefulness and decreases during sleep, particularly in response to prolonged wake states, suggesting a potential role in sleep pressure accumulation and discharge [64]. However, the extent to which astrocytes directly drive sleep–wake transitions versus supporting neuronal circuits in regulating these states remains an open question.

Ongoing research into the morphological, molecular and functional heterogeneity of astrocytes could further shed light on their specific implications for sleep regulation, which will be crucial for deciphering their precise roles in neural network dynamics and sleep homeostasis [147–149].

### Technical and methodological challenges in astrocyte research

Despite the growing recognition of astrocytes in sleep regulation, significant methodological challenges persist in accurately dissecting their contributions. One major limitation is the difficulty in isolating astrocyte-specific functions due to their intricate interactions with neurons, microglia, and vascular cells. The use of genetic tools such as Cre-loxP systems, optogenetics, and chemogenetics has provided invaluable insights, but many of these approaches lack absolute specificity, as promoters commonly used in astrocytic transgenic models can also be active in neurons. This overlap complicates the interpretation of results and necessitates refined techniques to achieve selective astrocyte manipulation.

Additionally, the spatial and temporal resolution of current imaging technologies still presents challenges in capturing astrocytic dynamics in vivo. Genetically encoded calcium indicators and neurotransmitter sensors have improved our ability to visualize astrocytic activity, yet they remain limited in resolving fine-scale astrocyte-neuron interactions. The integration of advanced techniques such as single-cell transcriptomics, super-resolution microscopy, and spatial proteomics may offer deeper insights into astrocytic heterogeneity and their distinct functional roles in sleep.

Another key challenge is the interpretation of data from in vitro versus in vivo studies. While in vitro models enable precise manipulation of astrocytic pathways, they lack the complexity of intact neural networks, including interactions with neurons and the vascular system. Conversely, in vivo studies provide a physiologically relevant context but are constrained by the invasive nature of imaging and electrophysiological techniques. Given that astrocytes play regionally specific roles in synaptic and metabolic regulation, findings from one brain area may not be directly generalizable to others, emphasizing the need for context-specific experimental approaches.

### Implications for sleep pathologies and future research

Finally, although the role of astrocytes in sleep-associated pathologies is not addressed in this review, it is important to highlight that astrocytes are also contributing to neuroinflammation, phagocytosis, and reactive gliosis, processes intimately linked to sleep disorders and neurodegenerative diseases [150]. Their ability to modulate inflammatory cascades suggests that they may play a pivotal role in pathological sleep disturbances, including insomnia, hypersomnia, and sleep fragmentation in neurodegenerative conditions [150, 151].

As sleep disorders affect over a third of the population and pose a significant societal and health burden, understanding astrocytic contributions to sleep regulation is essential. Future research should aim to refine experimental strategies that improve astrocyte specificity while considering their interactions with other glial cells and neuronal circuits. Advancements in imaging techniques, multi-omics approaches, and targeted pharmacological interventions may pave the way for novel therapeutic strategies aimed at restoring sleep homeostasis through astrocytic modulation.

## Data Availability

The datasets supporting this study are available from the website of PubMed (https://pubmed.ncbi.nlm.nih.gov/).
